# Global DNA methylation profiles of buffalo (*Bubalus bubalis*) preimplantation embryos produced by handmade cloning and in vitro fertilization

**DOI:** 10.1038/s41598-022-09207-8

**Published:** 2022-03-25

**Authors:** Shivani Malpotra, Pallavi Goel, Songyukta Shyam, Manoj Kumar Singh, Prabhat Palta

**Affiliations:** Embryo Biotechnology Lab, Animal Biotechnology Centre, ICAR-National Dairy Research Institute (Deemed University), Karnal, Haryana 132001 India

**Keywords:** Biological techniques, Epigenetics analysis, Methylation analysis

## Abstract

Somatic cell nuclear transfer technique (SCNT) has proved to be an outstanding method of multiplication of elite animals but accompanied with low efficiency and live birth rate of cloned animals. Epigenetic alterations of DNA has been one of the culprits behind this issue. Cloned embryos are found to deviate slightly from regular pattern of demethylation and re-methylation at the time of nuclear reprogramming and embryonic development when compared with embryos produced by in vitro fertilization (IVF). Thus, the present study was aimed at evaluating global DNA methylation profiles of cloned embryos at 2-cell, 8-cell and blastocyst stages and compare it with corresponding stages of embryos produced by IVF by using MeDIP-Sequencing on Illumina-based platform. We found out that cloned embryos exhibited significantly different DNA methylation pattern as compared to IVF embryos with respect to distribution of differentially methylated regions in different components of genome, CpG islands distribution and methylation status, gene ontological profiles and pathways affected throughout the developmental stages. The data generated from MeDIP-Seq was validated at blastocyst stage cloned and IVF embryos by bisulfite-sequencing PCR on five randomly selected gene regions.

## Introduction

The field of embryology gained emphasis in scientific research with the success of somatic cell nuclear transfer (SCNT) technique. SCNT technique is based on converting a differentiated somatic cell to the totipotent state upon reprogramming of epigenetic marks by the factors present in the cytoplasm of the enucleated oocyte. Till date, more than 20 mammalian species have been cloned, using different type of somatic cells^1^, for multiple purposes including agriculture, biomedical industry, disease modelling, therapeutic cloning, bio-pharming, conservation and restoration of endangered species and xenotransplantation demonstrating the usefulness of this procedure^2^.

Despite successful cloning in several farm animal species, SCNT has not been implemented on a large scale primarily due to the very low live birth rate obtained with cloned embryos^1,3–6^. In comparison with the live birth rate obtained with bovine embryos produced by in vitro fertilization (IVF) which is over 40%^7^, the live birth rate obtained with cloned embryos is very low. In cattle, it is only 9%^3,8^ to 5–20%^1,9^ whereas, across species, the live birth rate obtained with cloned embryos is only 1–6%^4,9,10^. In buffalo too, the live birth rate is < 2%^11,12^. This is due to a multitude of reasons such as low conception rate, high abortion rates, prolonged gestation, high incidence of abnormalities such as large offspring syndrome, severe placental deficiency, respiratory problems, short life span and perinatal death^13–16^.

SCNT is a complex technique with several factors which can influence its outcome such as the quality of recipient oocyte in terms of its cytoplasmic volume and factors necessary for reprogramming donor nucleus^17–19^, origin, quality and plasticity of nucleus of donor somatic cell^20,21^ and technical skill-based and biological variations in different steps of cloning procedure^22^. Although the exact reasons behind the problems associated with cloning through SCNT are not fully understood, incomplete nuclear reprogramming is considered to be the principal reason responsible for the abnormalities in cloned embryos. Nuclear reprogramming is the process of reversing a differentiated somatic nucleus to the totipotent state of the embryo after nuclear transfer^23^. This involves changes in the expression profile of 10,000 to 12,000 genes which drive the embryonic and fetal development^24^. During nuclear reprogramming, the epigenetic marks specific to the differentiated somatic cell must be fully erased, and those associated with the totipotent stage of the embryo must be re-established. The epigenetic reprogramming after SCNT has been often found to be abnormal or aberrant^23^. Therefore, epigenetics plays a crucial role in determining the cloning efficiency and the fate of cloned offspring born.

DNA methylation is one of the major epigenetic marks that plays crucial role at the time of nuclear reprogramming of SCNT embryos. DNA methylation occurs predominantly at CpG dinucleotides and is involved in several key genome functions such as imprinting, X-chromosome inactivation, genome stability, silencing of retrotransposons and inactivation of cancer-related genes^25,26^. Basically, DNA methylation suppresses gene expression by recruiting methyl- CpG-binding proteins such as MECP2 (methyl-CpG-binding protein 2), MBD1 (methyl-CpG-binding domain protein 1), MBD2, and MBD3, as well as associated histone deacetylases, co-repressor proteins and chromatin remodelling machineries at the promoter regions of specific genes^27^. DNA methylation is necessary for transcription of octamer-binding transcription factor 4 (OCT4) which is critical factor for pluripotency thus, certifies initiation and maintenance of early embryonic development after successful cloning^28,29^. Therefore, aberrant DNA methylation may cause abnormal gene expression during initial stages of development, ultimately leading to embryo/fetal developmental failure^23,30–32^.

Several studies have been made to investigate DNA methylation profile in SCNT embryos and compare it with their IVF counterparts. Umpteen of studies has been done with immunofluorescence technique to show aberrant pattern of DNA methylation in cloned embryos in many species. Dean et al.^33^ used indirect immunofluorescence method and observed that cloned mouse embryos showed reduction in methylation only up to one-cell stage. Also, pattern of DNA methylation of many cloned embryos was found to be similar to donor fibroblast cells. Santos et al.^34^ reported genome-wide failure of nuclear reprogramming in cloned bovine pre-implantation embryos at blastocyst stage using immunofluorescence. H3-K9 and DNA hypermethylation was found to be the major reason behind this failure. Likewise, aberrant DNA methylation pattern was observed along with variable methylation levels among nuclei within swamp buffalo SCNT embryos^35^. Histone hyperacetylation was also observed at 4- and 8-cell stage of SCNT embryos compared with IVF counterparts. Bisulfite sequencing was further comprehensively used to establish genome-wide methylation in cloned embryos. DNA methylation of bovine alpha satellite I repeat sequence was found to hypermethylated in blastocyst as well as in SCNT foetuses in comparison with in-vivo produced embryos^36^. Couldrey et al.^37^ reported repetitive satellite I sequence was found to differ significantly with regards DNA methylation and cell type (inner cell mass or trophectoderm) between cloned and in-vivo produced embryos. Likewise, comprehensive genome-wide methylome was also studied by applying several other Next Generation Sequencing (NGS) strategies such as Whole Genome Bisulfite Sequencing (WGBS)^38–40^ and Reduced Representation Bisulfite Sequencing (RRBS)^41,42^. To our information, there is only one report available on genome-wide DNA methylation profile of SCNT embryos in farm animals^43^. However, there is no report till date on the global DNA methylation profiling examined using a high throughput NGS technique in embryos produced by SCNT and IVF in any species. Thus, in the present study, we carried out genome-wide DNA methylation profiling in buffalo embryos produced by handmade cloning (HMC) and in-vitro fertilization (IVF) by using methylated DNA immunoprecipitation combined with high-throughput sequencing (MeDIP-Seq). To our knowledge, this is the first report on global DNA methylation pattern of cloned embryos relative to IVF counterparts in buffalo.

## Results

Under this study, global DNA methylation profile of pre-implantation buffalo embryos at 2-cell, 8-cell and blastocyst stages, produced by HMC and IVF, was generated by MeDIP-Seq for comparing the differentially methylated regions (DMRs) between cloned and IVF embryos at each developmental stage.

### Production of cloned and IVF embryos

**Figure 1:**
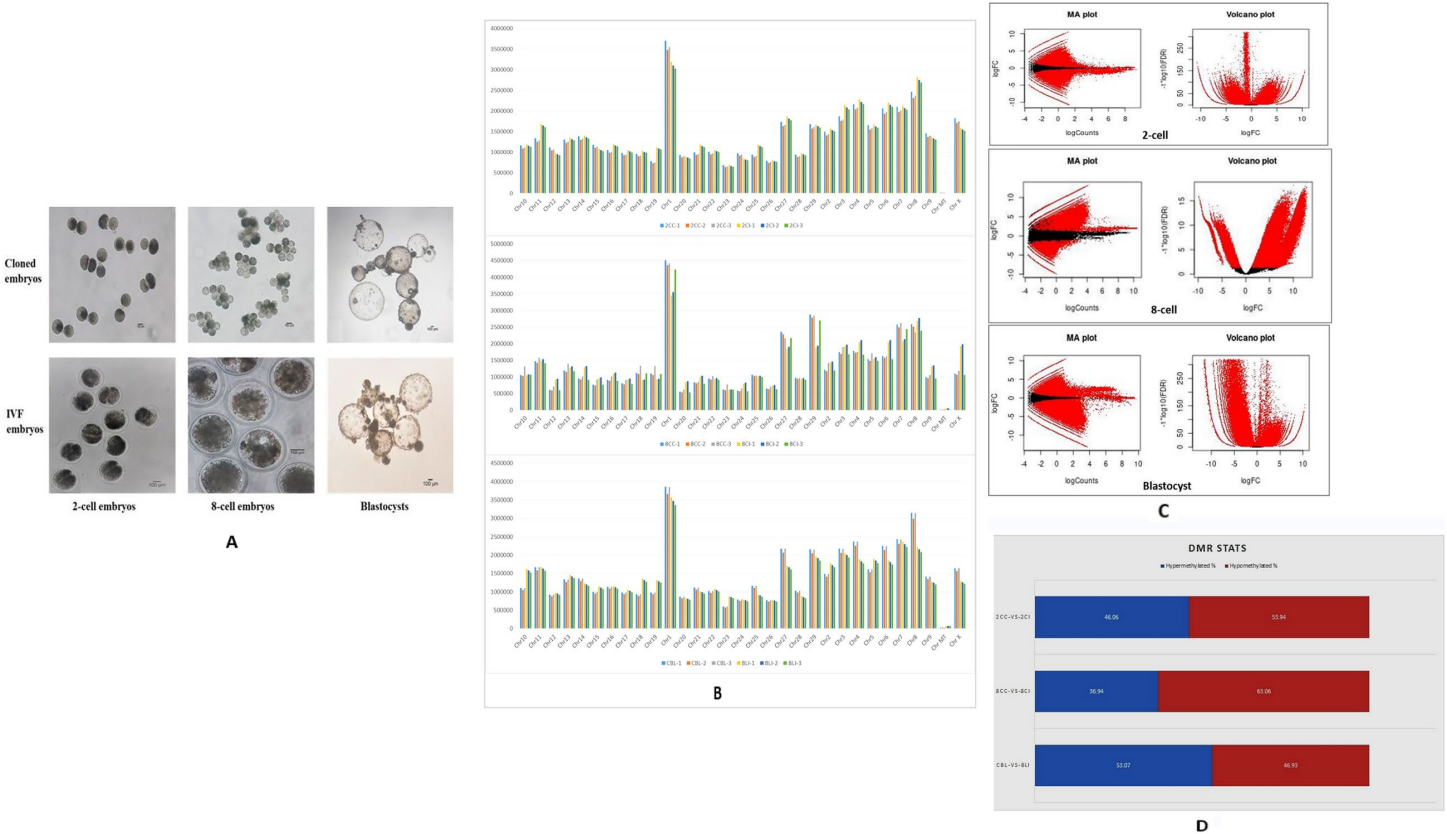
(**A**) Cloned and IVF embryos at 2-cell, 8-cell and blastocyst stage. (**B**) Reads of 2-cell stage cloned (2CC-1, 2CC-2 and 2CC-3) and IVF (2Cl-1, 2Cl-2 and 2Cl-3) embryos, 8-cell stage cloned (8CC-1, 8CC-2 and 8CC-3) and IVF (8CI-1, 8CI-2 and 8CI-3) embryos blastocyst stage cloned (BL-1, BL-2 and BL-3) and IVF (BLI-1, BLI-2 and BLI-3) embryos were mapped to different chromosomes. A maximum number of reads were found to map on chromosome number 1 across all the developmental stages. (**C**) MA plot depicting the global methylation pattern of hyper- and hypomethylated CpGs in cloned relative to IVF embryos at each developmental stage. The Y-axis represents the log fold ratio (M) and the X-axis, the mean average of normalized counts A. Red dots represent differentially methylated regions (DMRs) having adjusted P-value above the threshold value whereas, black dots represent DMRs having P-value below the threshold.Volcano plot showing global methylation pattern of hyper- and hypomethylated CpGs in cloned realtive to IVF embryos. Red dots indicate CpGs with significant differential methylation pattern whereas, black dots indicate CpGs, the methylation pattern of which was non-significant in the two groups. The dots towards the left, right and top sides denote hypomethylated, hypermethylated and most significant differentially methylated CpGs, respectively. (**D**) Overall distribution statistics of differentially methylated regions (DMRs). The X-axis represents comparison between cloned and IVF embryos at each developmental stage i.e., 2CC vs 2CI; 8CC vs 8CI and CBL vs BLI. The Y-axis represents the number of DMRs.

In this experiment, somatic cells from an adult bull (Mu-4093) were used for the production of cloned embryos at 2-cell, 8-cell and blastocyst stages by HMC. Semen obtained from the same bull was used for the production of IVF embryos at each of these stages (Fig. 1A). This was done to minimize the genetic variability between the cloned and IVF embryos since the embryos of the two groups produced in this manner were genetically half-identical. Blastocyst rate for cloned embryos varied from 30 to 35% for 15 trials whereas cleavage rate for 2-cell and 8-cell developmental stage was recorded to be 95–98% and 70–80% respectively. Likewise, for IVF embryos, blastocyst rate was observed to vary from 10–15% rate for 10 trials with cleavage rate of 15–22% was observed for 2- and 8-cell stages.

It has been reported that poor quality and low developmental competence of oocytes lead to lower cloning and IVF efficiency in terms of blastocyst rate and quality of embryos^44^. Therefore, it is necessary that competent and good quality oocytes are used for the production of cloned and IVF embryos that can be subjected to MeDIP-Seq. We achieved this by two means. Firstly, we selected only those embryos which appeared to be morphologically normal in appearance. Second, we selected oocytes of high developmental competence for cloning and IVF by staining them with Brilliant Cresyl Blue (BCB). It has been shown in a previous study in our laboratory that the blastocyst rate was higher for BCB + (oocytes with high developmental competence) than for BCB- (oocytes with low developmental competence) oocytes. Moreover, the blastocysts produced from BCB + oocytes had inner cell mass (ICM) cell number, ICM/trophectoderm (TE) cell number ratio, global level of H3K18ac, apoptotic index and expression level of *BCL-XL* similar to that of blastocysts produced through IVF^45^. Therefore, BCB + blastocysts had better developmental competence and were closer to IVF blastocysts in terms of quality, epigenetic status and gene expression than BCB- blastocysts, In view of this, only BCB + oocytes were used for HMC and IVF in the present study.

### Global DNA methylation of cloned and IVF buffalo embryos

In the present study, 130 GB MeDIP-Seq data was generated from three developmental stages (2-cell, 8-cell and blastocyst stages) of buffalo embryos produced by HMC and IVF. The number of total raw reads generated for cloned embryos (at 2-cell, 8-cell and blastocyst stage) was 466,173,180, whereas, the corresponding number for IVF embryos was 441,935,854. For all the samples, 86 to 94.4% of the total reads got aligned against the reference genome of *Bos taurus*, UMD 3.1.1 (Supplementary Fig. 1). The alignment statistics of the three biological replicates each of cloned and IVF embryos are given in (Supplementary Table 1).

#### Chromosomal distribution of MeDIP-Seq reads

The chromosomal distribution of the three biological replicates each of cloned and IVF 2-cell, 8-cell and blastocyst stage embryos is given in (Fig. 1B). MeDIP-Seq raw reads were detected in most of chromosomal regions (chromosome 1–29 and chromosome X) at the 2-cell, 8-cell and blastocyst stage in both cloned and IVF embryos. Maximum number of reads was found to map on chromosome number 1 as chromosome 1 is the largest chromosome in *Bos taurus* genome.

#### MA and volcano plot of cloned vs IVF embryos at different developmental stages

MA plot is commonly used to visualize the log fold change (FC) versus mean expression between two samples. It is a type of 2D scatter plot which allows visual display of base-2 log FC along the Y-axis and normalized mean expression along the X-axis, where each data point represents single CpG site. In the present study, this plot has been used to visualize log FC versus mean expression between cloned relative to IVF embryos at different developmental stages. Each data point with extreme values on the Y-axis represents the CpGs with significant differential methylation. Data points falling above the 1 threshold on the Y-axis indicate significant number of CpGs which were hypermethylated whereas, those below − 1, indicate CpGs which were hypomethylated. The data points falling close to 0 on the Y-axis indicates that both the samples share highly similar methylation pattern. Volcano plot is another type of scatter plot which allows quick visual identification of those data points (CpGs, genes etc.) that display changes of high magnitude which are statistically significant on plotting the measure of statistical significance (p-value) against the magnitude of the change (FC). In the present study, this scatter plot has been used to predict the status of CpGs which are significantly differentially methylated, either hyper- or hypomethylated in cloned relative to IVF embryos at given stages of cloned and IVF embryos (Fig. 1C). The CpGs sites seen dispersed towards the right side represent the hypermethylated sites, and those seen dispersed towards the left side represent the hypomethylated sites. The CpGs seen towards the top are those which are statistically most significantly differentially methylated.

#### Overall distribution of differentially methylated regions (DMRs)

The percentage of distribution of DMRs throughout the three developmental stages examined i.e., 2-cell, 8-cell, and blastocyst stages in cloned and IVF embryos is given in (Fig. 1D). In the cloned embryos, the number of highly methylated regions (HMRs) which was found to be higher at the 2-cell stage, decreased sharply at the 8-cell stage and then increased to the highest level at the blastocyst stage. In IVF embryos, number of HMRs increased from the 2-cell stage to the 8-cell stage and then declined sharply at the blastocyst stage. Also, distribution of HMRs in cloned 2-cell and 8-cell stage embryos was significantly (padj ≤ 0.05) less in comparison with IVF counterparts. The blastocyst stage of cloned embryos showed significantly (padj ≤ 0.05) higher numbers of HMRs. These results suggest that DNA methylation is altered dynamically throughout the developmental stages in cloned embryos in comparison with their IVF counterparts.

#### Determination of DMRs at each developmental stages

Total number of DMRs were detected at each developmental stage of cloned and IVF embryos. The number of uniquely present DMRs in cloned and IVF embryos at 2-cell stage was found to be 90,501 (11.2%) and 1,49,925 (18.6%), respectively. From among the commonly present DMRs i.e., 5,66,734, a total of 2,81,276 were found to be hypermethylated in cloned relative to IVF embryos. The number of uniquely present DMRs in cloned and IVF embryos at 8-cell stage was found to be 68,826 (14.7%) and 1,78,171 (38%), respectively. From among the commonly present DMRs i.e., 2,21,383, a total of 1,04,174 were found to be hypermethylated in cloned relative to IVF embryos.

**Figure 2:**
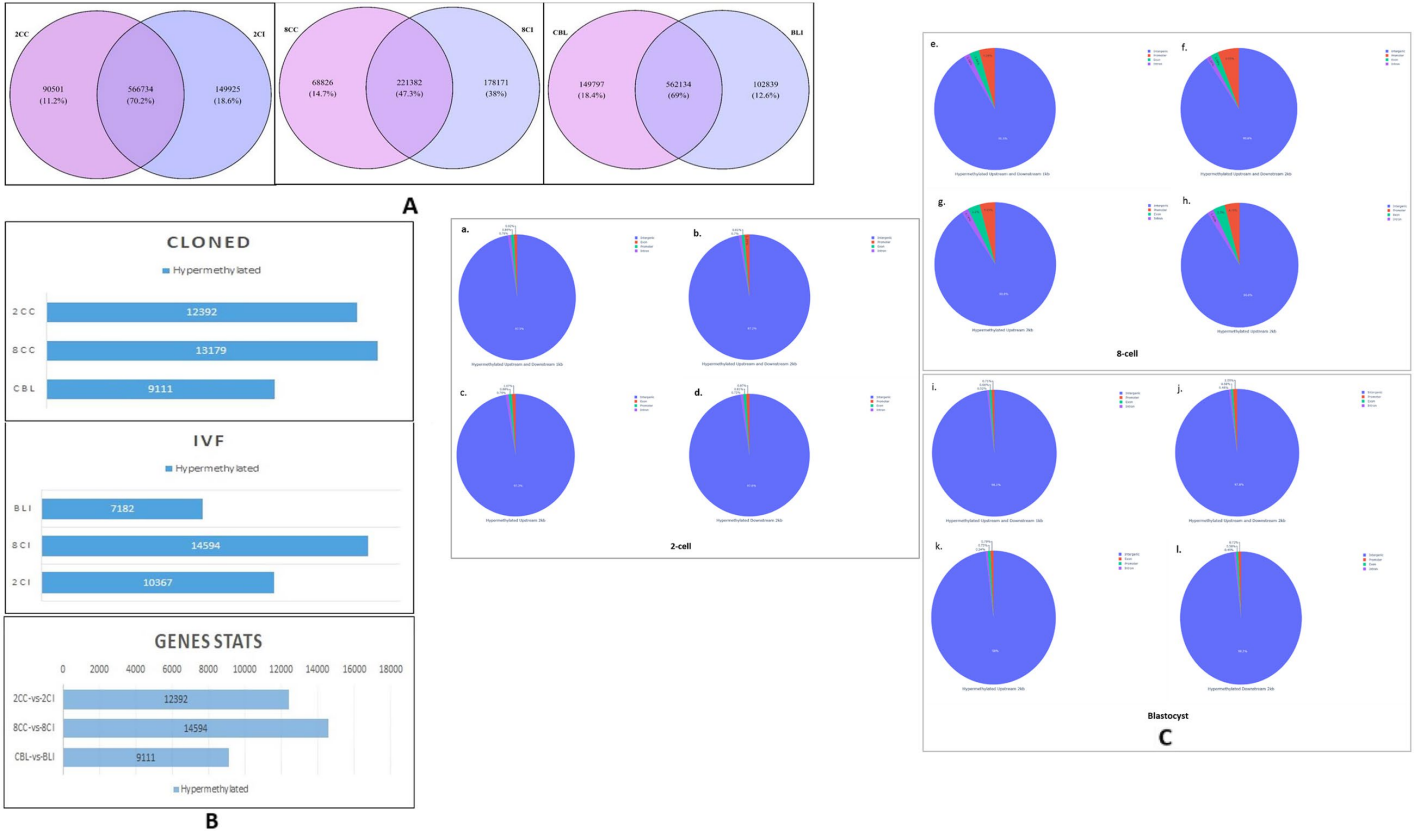
(**A**) Venn diagram depicting the DMRs hypermethylated commonly and uniquely in cloned and IVF 2-cell stage embryos, 8-cell stage and blastocyst stage embryos (P < 0.05). (**B**) Overall distribution statistics of methylation at gene level. The Y-axis represents cloned (2CC, 8CC and CBL) and IVF (2CI, 8CI and BLI) embryos and comparison between cloned and IVF embryos at different developmental stages (2CC vs 2CI, 8CC vs 8CI and CBL vs BLI). The X-axis represents the number of genes hypermethylated. (**C**) Venn diagram showing the overall genomic distribution of DMRs in cloned relative to IVF embryos. For 2-cell stage, (a and b) represent percentage of hypermethylated DMRs in upstream and downstream 1 kb and upstream and downstream 2 kb and (c and d) represent percentage of hypermethylated DMRs in upstream 2 kb and downstream respectively. For 8-cell stage, (e and f) represent percentage of hypermethylated DMRs in upstream and downstream 1 kb and upstream & downstream 2 kb and (g and h) represent percentage of hypermethylated DMRs in upstream 2 kb and downstream 2 kb respectively. For blastocyst stage, (i and j) represent percentage of hypermethylated DMRs in upstream and downstream 1 kb and upstream and downstream 2 kb and (k and l) represent percentage of hypermethylated DMRs in upstream 2 kb and downstream 2 kb respectively, of different genomic regions.

The number of uniquely present DMRs in cloned and IVF embryos was 1,49,797 (18.4%) and 1,02,839 (12.6%) respectively, whereas, 5,62,134 were commonly expressed in the two groups. From among the commonly present DMRs 2,82,582 were hypermethylated in cloned relative to IVF embryos. The Venn diagrams showing the DMRs commonly present in cloned and IVF 2-cell, 8-cell and blastocyst stage embryos and the DMRs uniquely present in the two groups are presented in (Fig. 2A).

#### Distribution of DMRs at gene level

Genes which overlap with HMRs in the promoter or gene-body regions are considered as methylated genes. If for HMRs coverage, edge R. logFC =  + 1 then the gene is designated as hypermethylated and if edge R. logFC = − 1, it is designated as unmethylated. In the present study, overall 27,518 genes were identified at the 2-cell stage, out of which 12,392 genes were found to be hypermethylated in cloned relative to IVF embryos. In case of 8-cell stage embryos, the total number of genes was found to be 23,621 of which, 14,594 genes were hypermethylated. Similarly, at the blastocyst stage, 24,556 genes were identified out of which, 9111 genes were hypermethylated in cloned relative to IVF embryos (Fig. 2B), Supplementary Table 2). The maximum number of hypermethylated genes was found at the 8-cell stage whereas, the minimum number was observed at the blastocyst stage. The number of genes showing hypermethylation at 2-cell, 8-cell and blastocyst stage in cloned and IVF embryos is shown in (Supplementary Table 3).

#### Distribution of DMRs in different components of the genome

Genome-wide DNA methylation profile in cloned and IVF embryos was observed with the distribution of MeDIP-Seq reads in different component of genome including upstream and downstream 1 kb and 2 kb promotor, exons, introns, intergenic region, CpG islands and repetitive elements.

Out of the 8,07,160 DMRs evaluated (P < 0.05), 3,71,777 DMRs were found to be hypermethylated in cloned relative to IVF 2-cell stage embryos. Of 4,68,379 DMRs evaluated (P < 0.05), 1,73,000 DMRs were found to be hypermethylated in cloned relative to IVF 8-cell stage embryos. Out of the 8,14,770 DMRs evaluated (P < 0.05), 4,32,379 DMRs were found to be hypermethylated in cloned relative to IVF blastocyst stage embryos. Major part of hypermethylated DMRs found at 2-, 8-, and blastocyst stages belong to intergenic region at upstream and downstream 1 kb and 2 kb, respectively. Venn diagram showing the distribution of hypermethylated DMRs in different genomic regions (promoter, exon, intron and intergenic) in cloned relative to IVF 2-, 8-cell and blastocyst stage embryos is presented in (Fig. 2C). The number and percentage of hypermethylated DMRs in upstream and downstream 1 kb and 2 kb, upstream 2 kb and downstream 2 kb in cloned relative to IVF embryos at each developmental stage is presented in (Supplementary Table 4) respectively.

#### CpG islands in cloned and IVF embryos

**Figure 3:**
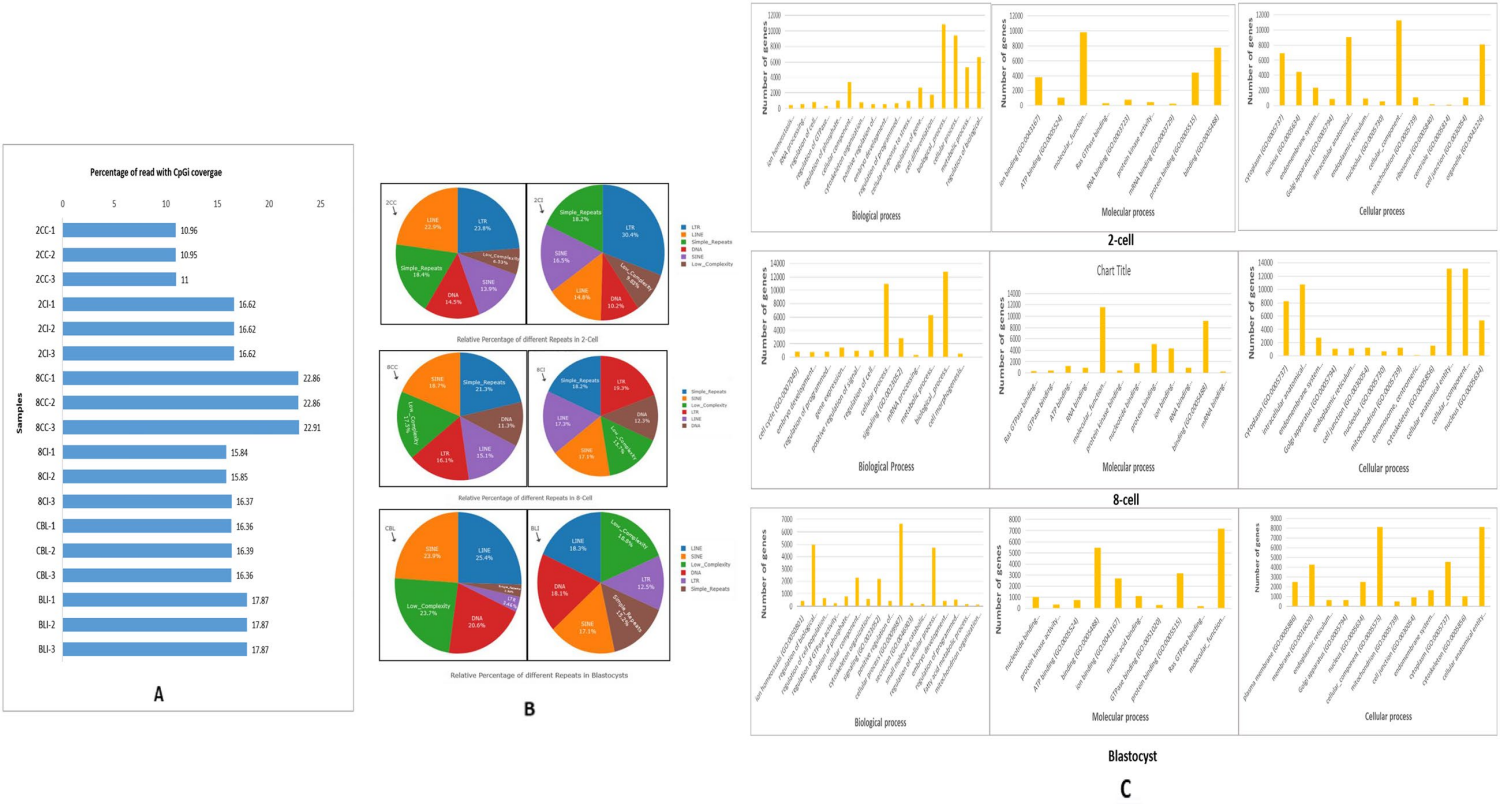
(**A**) Percentage of reads mapped with CpG islands for each sample. The X-axis represents percentage (%) range of mapped CGIs and the Y-axis represents different stages of buffalo cloned and IVF embryos with three biological replicates of each stage. (**B**) Pie-charts representing relative percentage of CpG islands found in a particular repetitive element at each developmental stage in cloned and IVF embryos relative to *Bos taurus* reference genome, UMD 3.1.1. (**C**) GO categories for Biological Process, Molecular process and Cellular process enriched across the hypermethylated genes in cloned relative to IVF 2-cell, 8-cell and blastocyst stage embryos.

Analysis of the percentage of reads which mapped distinctly with CpGs islands (CGIs) in all the biological replicates of cloned and IVF embryos at 2-cell, 8-cell and blastocyst stage showed a range of 10.95% to 22.91% (Fig. 3A). Maximum percentage of reads which mapped with CGIs for three biological replicates of cloned and IVF embryos at 2-cell stage was 11% and 16.62%, respectively. Similarly, for 8-cell stage cloned and IVF embryos, it was found to be 22.91% and 16.37%, whereas, in case of blastocysts, the corresponding values were 16.39% and 17.87%, respectively. In the present study, the total number of CpG islands distributed in buffalo embryo genome were observed. For the first time, we identified total 2,17,246 CpG islands, out of which 1,04,188 (42.55%) and 1,13,058 (48.07%) islands were present in cloned and IVF embryos, respectively. In the cloned embryos, 52,168 (23.72%) were found to be hypermethylated, whereas, the corresponding figures in IVF embryos were 41,365 (30.25%). The number and percentage of CpG islands hypermethylated in cloned versus IVF embryos at each developmental stage (i.e., 2CC vs 2CI, 8CC vs 8CI, CBL vs BLI) is given in (Table 1).

**Table 1:** Number of CpG islands showing hypermethylation in 2-cell, 8-cell and blastocyst stage cloned and IVF embryos.

Group	Methylation	Number of CpG islands	% of total CpGs islands
Cloned 2-cell	Hypermethylation	8290	2.23
Cloned 8-cell	Hypermethylation	32,715	18.91
Cloned blastocyst	Hypermethylation	11,163	2.58
IVF 2-cell	Hypermethylation	5895	2.1
IVF 8-cell	Hypermethylation	25,745	24.71
IVF blastocyst	Hypermethylation	9725	3.44
2CC-vs-2CI	Hypermethylation	8290	2.23
8CC-vs-8CI	Hypermethylation	32,715	18.91
CBL-vs-BLI	Hypermethylation	11,163	2.58

#### CpGs enrichment in cloned and IVF embryos

The assessment of CpGs enrichment value of methylated regions (frequency of CpGs wise) was done by dividing the relative frequency of CpGs (region.relH) of the regions by the relative frequency of CpGs (genome.relH) of the reference genome. Similarly, the final value of CpGs enrichment of methylated regions (ratio CpG wise) was done by dividing the observed/expected ratio of CpGs within the region (region.goge) by the observed/expected ratio of CpGs within the reference genome (genome.goge). The enrichment score for both frequency CpGs and ratio CpGs in the genomic regions sequenced compared to the reference genome was above 1 in all the cases (Table 2), indicating a successful enrichment of methylated fragments in the data sets.

**Table 2:** CpG enrichment of methylated regions in 2-cell, 8-cell and blastocyst stage of cloned and IVF embryos.

Group	Relative frequency of CpGs within the regions. (Region.reIH)	Observed/expected ratio of CpGs within the regions (Region.goge)	Ratio of region.relH/genome.relH (enrichment. score.relH)	Ratio of region.goge/genome.goge (enrichment. score.goge)
Cloned 2-cell	2.262895	0.4060407	2.194193	1.695227
Cloned 8-cell	2.993334	0.4425746	2.902455	1.847757
Cloned blastocyst	3.831625	0.546323	3.715295	2.280909
IVF 2-cell	2.899797	0.4578469	2.811758	1.911519
IVF 8-cell	3.156285	0.4815534	3.060459	2.010494
IVF blastocyst	2.932217	0.4758824	2.843193	1.986818

#### Distribution of CpG islands in repetitive elements of genome

Distribution of CpG islands with hypermethylation in the repetitive elements of genome i.e., LINE, SINE, repetitive DNA, LTR, simple repeats and low complexity repeats etc. was examined between cloned and IVF embryos at different developmental stages and between different developmental stages (Supplementary Table 5). Relative percentage of CpG islands found in particular repeats in comparison with reference genome (UMD 3.1.1) at different developmental stages in cloned and IVF embryos is presented in (Fig. 3B). It was observed that at 2-, 8-cell and blastocyst stage cloned embryos, maximum distribution of CpGis was found in LTRs, simple repeats and LINE respectively.

### Gene ontology (GO) analysis

GO analysis was done in order to explore the biological significance, detailed annotation of gene function, biological process and cellular distribution of differentially methylated genes between cloned and IVF embryos. GO analysis is based on a controlled vocabulary of terms of three domains—“Biological Process” (BP), “Molecular Function” (MF) and “Cellular Compartment” (CC)^46^. Using GO terms, MeDIP-Seq results can be summarized to provide insights into the methylation status of genes between cloned and IVF embryos. In the present study, genes with hypermethylation (Edge R. Log fold change ≥  + 1) in cloned relative to IVF embryos were used for GO analysis.

A total of 483 Biological Process, 85 Molecular functions and 121 cellular components were found to be affected at 2-cell stage cloned embryos relative to IVF counterparts. GO analysis of differentially methylated genes in cloned and IVF 8-cell stage embryos revealed a total of 526 Biological Processes, 97 Molecular functions and 115 cellular components were found to be affected with DNA hypermethylation. Likewise, a total of 594 Biological Process, 131 Molecular functions and 176 Cellular components were affected in blastocyst stage cloned embryos relative to IVF ones. Among all three stages of cloned embryos relative to IVF counterparts, molecular function, binding, and protein binding were majorly affected in case of Molecular function GO term category. Cellular Component GO term including cell part, cellular component, and intracellular anatomical structure being the most enriched ones (Fig. 3C). The functional annotations, which were found to be hypermethylated are given in (supplementary Tables 6, 7, 8).

### Pathways analysis

At 2-cell stage cloned embryos, total 30 pathways were detected, out of which 15 pathways were affected by hypermethylation of genes related to embryonic development relative to IVF counterparts. Out of 28 pathways detected, 15 pathways were affected by hypermethylation of genes related to embryonic development in 8-cell stage cloned relative to IVF embryos. At blastocyst stage cloned embryos, 37 pathways were found, of which 27 pathways were affected by hypermethylation of genes related to embryonic development relative to IVF embryo (Table 3). Wnt signaling pathway, inflammation mediated by chemokine and integrin signaling pathway and gonadotropin-releasing hormone receptor pathway were majorly affected by hypermethylation of DNA throughout the developmental stages.

**Table 3:** Pathways affected by hypermethylation of genes in cloned relative to IVF at 2-cell, 8-cell and blastocyst stage embryos.

Pathways affected by hypermethylation	No. of genes	P value	FDR
**2-cell stage**			
Integrin signalling pathway (P00034)	162	7.13E−05	5.95E−03
Inflammation mediated by chemokine and cytokine signaling pathway (P00031)	197	5.62E−04	3.13E−02
Gonadotropin-releasing hormone receptor pathway (P06664)	193	8.59E−04	3.59E−02
PDGF signaling pathway (P00047)	120	2.15E−03	7.20E−02
CCKR signaling map (P06959)	140	2.28E−03	6.35E−02
Endothelin signaling pathway (P00019)	73	4.51E−03	1.08E−01
Apoptosis signaling pathway (P00006)	106	5.45E−03	1.14E−01
Ras Pathway (P04393)	62	1.13E−02	2.10E−01
Angiogenesis (P00005)	129	1.36E−02	2.27E−01
Wnt signaling pathway (P00057)	200	1.56E−02	2.36E−01
T cell activation (P00053)	72	2.19E−02	3.04E−01
EGF receptor signaling pathway (P00018)	111	2.35E−02	3.02E−01
B cell activation (P00010)	58	2.63E−02	3.14E−01
Heterotrimeric G-protein signaling pathway-Gq alpha and Go alpha mediated pathway (P00027)	91	3.82E−02	4.25E−01
Histamine H1 receptor mediated signaling pathway (P04385)	39	3.90E−02	4.07E−01
**8-cell stage**			
Angiogenesis (P00005)	165	4.38E−04	3.65E−02
Integrin signalling pathway (P00034)	177	5.97E−04	3.33E−02
PDGF signaling pathway (P00047)	139	1.99E−03	8.32E−02
CCKR signaling map (P06959)	158	4.36E−03	1.46E−01
Wnt signaling pathway (P00057)	236	8.09E−03	2.25E−01
Gonadotropin-releasing hormone receptor pathway (P06664)	207	1.27E−02	3.02E−01
Alzheimer diseasE−presenilin pathway (P00004)	110	1.59E−02	3.32E−01
Ras Pathway (P04393)	69	1.98E−02	3.67E−01
EGF receptor signaling pathway (P00018)	127	3.11E−02	5.19E−01
Endothelin signaling pathway (P00019)	76	3.49E−02	5.30E−01
Apoptosis signaling pathway (P00006)	113	3.63E−02	5.05E−01
Oxytocin receptor mediated signaling pathway (P04391)	59	4.03E−02	5.17E−01
PI3 kinase pathway (P00048)	56	4.48E−02	5.34E−01
Oxidative stress response (P00046)	56	4.48E−02	4.99E−01
5HT2 type receptor mediated signaling pathway (P04374)	67	4.72E−02	4.93E−01
**Blastocyst stage**			
Inflammation mediated by chemokine and cytokine signaling pathway (P00031)	156	8.20E−05	6.85E−03
Wnt signaling pathway (P00057)	168	2.71E−04	1.51E−02
Endothelin signaling pathway (P00019)	62	4.57E−04	1.91E−02
Heterotrimeric G-protein signaling pathway-Gq alpha and Go alpha mediated pathway (P00027)	83	4.92E−04	1.64E−02
Integrin signalling pathway (P00034)	115	9.68E−04	2.69E−02
Blood coagulation (P00011)	44	2.10E−03	5.00E−02
Nicotinic acetylcholine receptor signaling pathway (P00044)	66	2.52E−03	5.25E−02
PDGF signaling pathway (P00047)	90	3.24E−03	6.02E−02
Angiogenesis (P00005)	102	4.10E−03	6.85E−02
DNA replication (P00017)	4	5.33E−03	8.09E−02
Ionotropic glutamate receptor pathway (P00037)	39	5.61E−03	7.81E−02
Heterotrimeric G-protein signaling pathway-Gi alpha and Gs alpha mediated pathway (P00026)	99	7.23E−03	9.29E−02
Alzheimer diseasE−amyloid secretase pathway (P00003)	45	8.11E−03	9.67E−02
EGF receptor signaling pathway (P00018)	86	1.19E−02	1.32E−01
Nicotine pharmacodynamics pathway (P06587)	28	1.28E−02	1.34E−01
Gonadotropin-releasing hormone receptor pathway (P06664)	134	1.31E−02	1.29E−01
Metabotropic glutamate receptor group I pathway (P00041)	21	1.37E−02	1.27E−01
Alzheimer disease-presenilin pathway (P00004)	73	1.44E−02	1.27E−01
Histamine H1 receptor mediated signaling pathway (P04385)	32	1.57E−02	1.31E−01
Metabotropic glutamate receptor group III pathway (P00039)	46	1.98E−02	1.58E−01
Cadherin signaling pathway (P00012)	70	3.19E−02	2.42E−01
5HT2 type receptor mediated signaling pathway (P04374)	45	3.34E−02	2.42E−01
Axon guidance mediated by netrin (P00009)	26	3.51E−02	2.44E−01
Oxytocin receptor mediated signaling pathway (P04391)	39	3.64E−02	2.43E−01
Insulin/IGF pathway-protein kinase B signaling cascade (P00033)	27	4.45E−02	2.86E−01
B cell activation (P00010)	42	4.73E−02	2.92E−01
Plasminogen activating cascade (P00050)	17	4.94E−02	2.95E−01

### Methylation status of important genes involved in embryonic development in cloned relative to IVF embryos

Methylation status of some important genes involved in embryonic development viz. pluripotency-related genes, imprinted genes, apoptosis-related genes, cell cycle-related genes, methylation-specific genes and some functionally important genes is given in (Table 4). A particular gene is depicted as hypermethylated if it overlaps with highly methylated region (HMR) even in a single gene region. However, the actual genes expression level of a particular gene can vary irrespective of presence of methylation in any region of that gene. There is a complex relationship between DNA methylation and gene expression as higher gene expression levels are generally linked with low methylation at promotor region but higher gene body methylation^47^. The exact expression status of a gene can be known only after examining the correlation of DNA methylation with mRNA expression profile of that particular gene.

**Table 4:** Methylation status of genes related to embryonic development in blastocyst stage cloned embryos relative to IVF counterparts.

Gene symbol	Gene name	Chromosomal location	No. of regions hypermethylated at		
			2-cell stage	8-cell stage	Blastocyst-stage
*POU2F1*	POU domain, class 2, transcription factor 1	GK000003.2	12	03	03
*NANOG*	Nanog homeobox	GK000005.2	0	01	0
*KLF4*	Kruppel-like factor 4	GK000008.2	02	03	0
*TCF3*	Transcription factor 3 (E2A immunoglobulin enhancer-binding factors E12/E47)	GK000007.2	04	07	02
*LIN28b*	Lin-28 homolog B	GK000009.2	07	0	07
*TDGF1*	Teratocarcinoma-derived growth factor 1	GK000002.22	02	01	0
*SALL4*	Spalt like transcription factor 4	GK000001.23	06	08	0
*DUSP1*	Dual specificity protein phosphatase 1	GK000001.29	22	43	06
*DUSP6*	Dual specificity protein phosphatase 6	GK000005.2	0	03	0
*FGF4*	Fibroblast growth factor 4	GK000002.29	0	0	0
*GRB10*	Growth factor receptor-bound protein 10	GK000004.2	18	22	03
*PEG10*	Paternally expressed gene 10	GK000004.2	02	02	01
*MEST*	Mesoderm specific transcript	GK000004.2	02	02	01
*IGF2R*	Insulin-like growth factor 2 receptor	GK000009.2	17	28	03
*MAOA*	Monoamine oxidase A	GK000030.2	10	04	08
*GNAS*	Guanine nucleotide-binding protein	GK000001.23	07	14	0
*NDN*	Necdin	GK000002.21	0	01	0
*PHLDA2*	Pleckstrin homology like domain family A member 2	GK000002.29	0	0	0
*CDKN1C*	Cyclin dependent kinase inhibitor 1C	GK000002.29	0	02	0
*PLAG1*	Pleomorphic adenoma gene 1	GK000001.24	0	01	0
*ASCL2*	Achaete-scute complex homolog 2	GK000002.29	0	01	0
*CASP9*	Caspase 9	GK000001.26	02	03	03
*BAX*	Bcl-2-associated X protein	GK000001.28	01	03	0
*BAD*	BCL2-antagonist of cell death	GK000002.29	01	06	0
*BCL2L1*	BCL2-like 1	GK000001.23	06	22	0
*DFFA*	DNA fragmentation factor subunit alpha	GK000001.26	01	04	02
*FAS*	Fas cell surface death receptor	GK000002.26	03	05	04
*APAF1*	Apoptotic protease activating factor 1	GK000005.2	10	03	02
*HSPA1A*	Heat shock protein family A (Hsp70) member 1A	GK000002.23	01	02	0
*PTEN*	Phosphatase and tensin homolog	GK000002.26	19	03	03
*MDM2*	Mouse double minute 2 homolog	GK000005.2	03	05	01
*CDK4*	Cyclin dependent kinase 4	GK000005.2	0	03	0
*NPAT*	Nuclear protein, coactivator of histone transcription	GK000001.25	05	01	01
*DNMT1*	DNA methyltransferase 1	GK000007.2	05	06	0
*DNMT3A*	DNA methyltransferase 3A	GK000001.21	01	12	0
*DNMT3B*	DNA methyltransferase 3B	GK000001.23	05	06	0
*TET1*	Tet methylcytosine dioxygenase 1	GK000002.28	27	32	0
*TET2*	Tet methylcytosine dioxygenase 2	GK000006.2	10	02	03
*TET3*	Tet methylcytosine dioxygenase 3	GK000001.21	07	21	01
*EZH2*	Enhancer of zeste 2 polycomb repressive complex 2 subunit	GK000004.2	08	06	0
*MBD3*	Methyl-CpG binding domain protein 3	GK000007.2	02	01	0
*YY1*	Yin Yang 1	GK000002.21	04	12	0
*TIMP2*	TIMP metallopeptidase inhibitor 2	GK000001.29	03	09	02
*BMP7*	Bone morphogenetic protein 7	GK000001.23	07	03	18
*AQP9*	Aquaporin 9	GK000001.20	02	0	03
*SOD1*	Superoxide dismutase 1	GK000001.2	02	04	0
*FGF7*	Fibroblast growth factor 7	GK000001.20	11	01	09
*IL6*	Interleukin 6	GK000004.2	02	02	0
*G6PD*	Glucose-6-phosphate dehydrogenase	GK000030.2	02	07	0
*MTPN*	Myotrophin	GK000004.2	08	20	01
*WNT2*	Wnt family member 2	GK000004.2	06	06	08
*DKK3*	Dickkopf WNT signaling pathway inhibitor 3	GK000001.25	07	05	09
*COL4A1*	Collagen type IV alpha 1 chain	GK000001.22	02	27	11
*TCF7*	Transcription factor 7	GK000007.2	02	06	03
*CRABP2*	Cellular retinoic acid binding protein 2	GK000003.2	0	02	02
*PPARA*	Peroxisome proliferator activated receptor alpha	GK000005.2	07	02	21
*PCGF2*	Polycomb group ring finger 2	GK000001.29	01	01	0
*MCL1*	Induced myeloid leukemia cell differentiation protein-1	GK000003.2	01	03	0
*DYSF*	Dysferlin	GK000001.21	10	11	53
*HDAC8*	Histone deacetylase 8	GK000030.2	30	20	03
*LEF1*	Lymphoid enhancer binding factor 1	GK000006.2	20	08	13
*IFN-τ*	Interferon tau	GK000008.2	02	09	0

### Results validation

**Figure 4:**
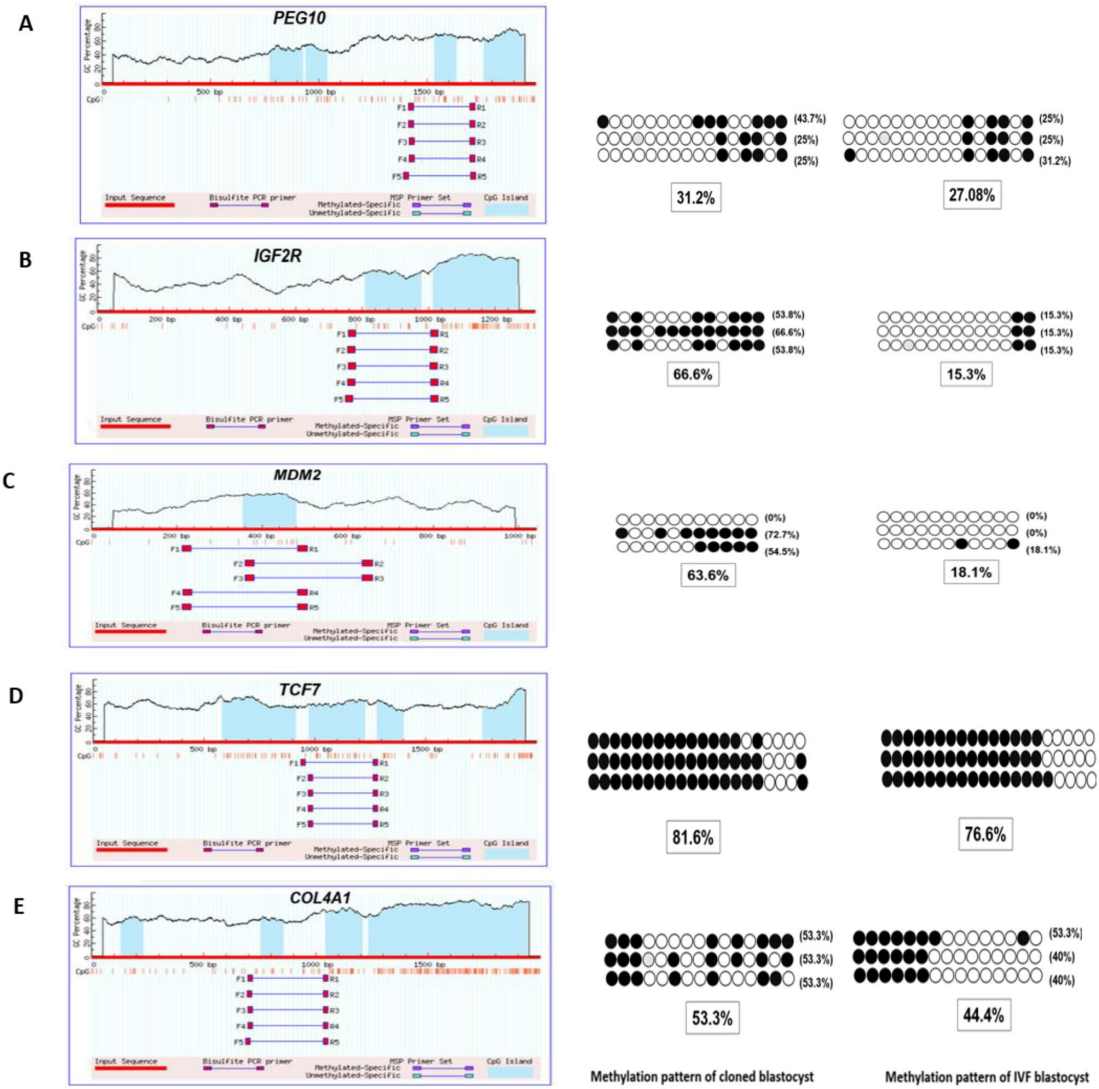
Validation of MeDIP-Seq data by bisulfite sequencing. A total of 5 genes were selected randomly to validate MeDIP-Seq data by bisulfite PCR in blactocyst-stage cloned and IVF embryos. CpG islands of upstream 2 k of all selected genes were predicted by MethPrimer. No of circles in each horizontal line represent no of CpGs present in a particular regions. Unfilled (white) and filled (black) circles represent unmethylated and methylated CpGs, respectively. Horizontal lines of circles represent one separate replicate that was sequenced. Lollipop diagrams were generated by BIQ Analyzer software. For each sample, the methylation data were analyzed by computing the percentage of methylated CpGs of the total number of CpGs. (**A**) DNA methylation status of upstream 2 kb of *PEG10*. F2-R2 region was chosen as the analysed region. 16 circles in each horizontal line represent 16 CpGs of *PEG10*. (**B**) DNA methylation status of upstream 2 kb of *IGF2R*. F2-R2 region was chosen as the analyzed region. 13 circles in each horizontal line represent 13 CpGs of IGF2 (**C**) DNA methylation status of upstream 2 kb of *MDM2*. F1-R1 regions was chosen as the analyzed region. 11 circles in each horizontal line represent 11 CpGs of *MDM2*. (**D**) DNA methylation status of upstream 2 kb of *TCF7*. F1-R1 region was chosen as the analyzed region. 20 circles in each horizontal line represent 20 CpGs of *TCF7*. (**E**) DNA methylation status of upstream 2 kb of *COL4A1*. F2-R2 region was chosen as the analyzed region. 15 circles in each horizontal line represent 15 CpGs of *COL4A1*.

To confirm the results of MeDIP-Seq data, five genes (*IGF2R, PEG10, MDM2, TCG7* and *COL4A1*) were selected randomly to perform bisulfite sequencing PCR (BSP). Primers were designed using MethPrimer software. The total of number of blastocysts used for bisulfite sequencing PCR in cloned and IVF groups was 25 for each group. Each sample was sequenced in triplicate. Data generated from Sanger sequencing was analysed by BioEdit version 7.2.5. All the five gene regions which had showed hypermethylation in cloned relative to IVF embryos in MeDIP-Seq data, showed hypermethylation in bisulfite sequencing PCR also which validated and confirmed the reliability of our data obtained from MeDIP-Seq (Fig. 4).

## Discussion

Although, some reports are available in bovine and other species on the DNA methylation in cloned compared to IVF embryos at different developmental stages, these reports provide a limited insight on the aberrations in DNA methylation in cloned embryos since low throughput techniques like immunostaining and RT-PCR were used for examining the methylation status in these studies^33–35^. To our information, there is no report in any species on the comparative global DNA methylation profile of cloned and IVF embryos at different developmental stages using a high throughput technique like MeDIP-Seq.

Over the years, many methods have been developed for analyzing genome-wide DNA methylome. These include whole genome bisulfite sequencing (WGBS), reduced representation bisulfite sequencing (RRBS), TET‑assisted bisulfite sequencing (TAB‑seq), comprehensive high‑throughput arrays for relative methylation (CHARM), methylation array and MeDIP-Seq. However, each method has its own merits and demerits when it comes to sequence whole genome. WGBS is considered to be the best method for examination of DNA methylation profile of mammalian genome due to its high resolution. But it is costly, time consuming and generates complicated data that further needs comprehensive bioinformatics analysis which limits the popularity of this technique. MeDIP-Seq is a widely used affinity-based method for examining DNA methylation. Global DNA methylation analysis is feasible with MeDIP-Seq even with very low amount of starting (as low as 1 ng) DNA sample^48,49^. Many recent studies have used MeDIP-Seq to generate relative genome-wide DNA methylation profile^50–52^. Therefore, we selected this method to study genome-wide DNA methylation in cloned embryos in comparison with IVF embryos in buffalo.

In the present study, DNA methylation profile of cloned and IVF embryos generated by MeDIP-Sequencing showed significant difference between both kind of embryos in reference to number and distribution of DMRs and CpGis in different parts of genome. Also, cloned embryos were found to be hypermethylated with respect to IVF counterparts which affected various important pathways involved in embryonic development. Hypermethylation of DNA throughout all the developmental stages studied in cloned embryos affected the methylation status of many functional, structural and imprinted genes that may lead to abnormal gene expression.

An unusual pattern of de-methylation and re-methylation in cloned embryos has been reported in several 5-methylcytosine immunofluorescence-based studies in past years^33–35,43^. Our study was not immunofluorescence-based but our MeDIP-Seq data is in agreement with the results of these low-throughput studies as cloned embryos showed hypermethylation of DNA at blastocyst stage in comparison to IVF counterparts.

Dean et al.^33^ reported that cloned bovine embryos lack passive demethylation mechanism and have high expression of *DNMT3A* and *DNMT3B* which is responsible for de novo methylation at 4-cell and 8-cell stages. Likewise, in the present study, 23,621 genes were identified at the 8-cell stage in cloned embryos out of which 14,594 showed hypermethylation. This scenario reflects re-methylation of genes due to re-establishment of methylation marks that coincides with time of embryonic genome activation (EGA). Memili et al.^53^ suggested the same possible reason for this pattern of methylation at 8-cell stage as this stage involves major change in transcriptional activation of the embryonic genome which might activate Dnmt3a and Dnmt3b enzymes.

Dean et al.^33^ reported increased methylation level in the bovine blastocyst stage embryos specifically in the trophectoderm cells. Contrary to this, we found that the less number of genes were showing hypermethylation (9111) in cloned embryos relative to IVF embryos at the blastocyst stage.

Using single-cell RRBS to generate DNA methylomes for SCNT embryos in mice, Liu et al.^54^ reported abnormally high levels of DNA methylation in 2-cell and 4-cell stage SCNT embryos. It was found that, SCNT embryos at 4-cell stage were relatively hypermethylated compared with 2-cell stage embryos which opposed the demethylation pattern of normal embryos. Also, the aberrant DNA methylation in arrested SCNT embryos was attributed to differences in expression of *Dnmt1* and *Tet1* enzymes. However, our study showed a slightly different trend as we found that 27,517 genes were identified in 2-cell stage cloned embryo, with 12,392 genes showing hypermethylation at 2-cell stage cloned embryos in comparison to IVF counterparts. This suggests that the majority of genes undergo demethylation at early developmental stages during nuclear reprogramming. In our data, *DNMT1* and *TET1* showed hypermethylation which could be the reason for lower methylation level of genes in 2-cell stage cloned embryos relative to their IVF counterparts. It is suggested that cytoplasmic factors of oocytes might help in triggering demethylation with remodelling of chromatin.

To our information, there is only one study in which genome-wide DNA methylation has been studied in SCNT preimplantation embryos but it is focused on specific parts of genome. Zhang et al.^43^ compared DNA methylation reprogramming in bovine SCNT and IVF preimplantation embryos and analyzed the influence of vitamin C (VC) on the reprogramming of DNA methylation by using bisulfite sequencing. The results showed that global DNA methylation followed a typical pattern of demethylation and remethylation in IVF preimplantation embryos however, the global genome remained hypermethylated in SCNT preimplantation embryos. Compared with the IVF group, pluripotency genes *POU5F1* and *NANOG* showed insufficient demethylation and hypermethylation in the SCNT group. Similarly, in our study, we found that pluripotency genes (*POU5F1, TCF3, LIN28b, and DUSP1*) showed high number of regions with hypermethylation which suggests lower expression of these genes.

Gene ontology analysis (GO) of differentially methylated genes in the present study showed 483 biological process, 85 molecular function and 121 cellular components to be affected by hypermethylation of genes in cloned 2-cell stage embryos relative to their IVF counterparts. Similarly, in the 8-cell stage cloned embryos, 526 biological process, 97 molecular function and 115 cellular components were found to be affected by hypermethylation of genes with respect to IVF embryos. GO analysis for cloned blastocyst-stage embryos relative to IVF embryos revealed 594 biological process, 131 molecular function and 176 cellular component to be affected by hypermethylation.

In the present study, 15 pathways were found to be affected at the 2-cell stage, 15 at the 8-cell stage and 27 at the blastocyst stage in cloned embryos relative to IVF counterparts. For all the developmental stages covered under present study, the major pathways affecting embryonic development in cloned embryos relative to IVF counterparts were found to be Wnt signaling pathway, inflammation mediated by chemokine and cytokine signalling pathways, gonadotropin-releasing hormone receptor pathway and Integrin signalling pathway. These pathways were affected by hypermethylation of DNA.

It was found in the present study that imprinting genes such as *IGF2R, PEG10, GRB10, MEST, and MAOA* showed presence of hypermethylated regions that suggest lower gene expression at the blastocyst stage in cloned relative to IVF embryos. Some important apoptosis-related genes such as *CASP9* and *DFFA, FAS, APAF1, and PTEN* were found to have hypermethylation in gene region in blastocyst stage cloned embryos compared to their IVF counterparts.

Similarly, cell cycle-related genes such as *MDM2,* and *NPAT* showed presence of hypermethylation in gene regions in blastocyst stage cloned embryos relative to IVF counterparts.

We found that the methylation status of methylation-specific genes viz. *DNMT1, DNMT3A, DNMT3B, TET1, EZH2,* and *MBD3* showed no methylation in gene regions with whereas *TET2 and TET3* gene found to have hypermethylation in gene regions in blastocyst stage cloned embryos relative to their IVF counterparts. This pattern of methylation reflects high expression of abovementioned genes in the cloned blastocyst-stage embryos which might be responsible for the abnormal hypermethylation observed in cloned blastocysts in comparison with IVF counterparts.

There are some functionally important genes which contribute to embryonic development in bovine embryos. We found that *YY1, SOD1, IL6, G6PD, PCGF2, MCL1,* and *IFN-τ* represented no methylation in the gene regions whereas *TIMP2, MTPN, COL4A1, TCF7, AQP9, BMP7, FGF7, WNT2, DKK3, CRABP2, PPARA, DYSF*, *LEF1 and HDAC8* showed high number of regions with hypermethylation in blastocyst stage cloned embryos compared to their IVF counterparts. Shyam et al.^55^ also observed a high expression of WNT signalling pathway genes i.e. *TCF7* and *LEF1* in cloned buffalo blastocyst-stage embryos. They also reported lower gene expression level of *IFN-τ* (primary maternal signal for recognition of pregnancy in ruminants) in cloned blastocyst was lower than in IVF counterparts.

Similarly, Sood et al.^56^ also reported down- regulated expression of *IFN-τ* in cloned embryos in comparison with IVF counterparts. In contrast, present study indicates hypermethylation of *TCF7* and *LEF1* at gene level which suggests lower expression of these genes in cloned embryos. Also, we found the gene region for *IFN-τ* to be unmethylated which points to a high expression of this gene in cloned compared to IVF embryos. Thus, our results regarding methylation status of *TCF7*, *LEF1* and *IFN-τ* genes were found to be not in agreement with above mentioned studies.

Kim et al.^57^ reported abnormally up-regulated expression of tissue inhibitor of metalloproteinase-2 (TIMP-2) and superoxide dismutase (SOD) in the placentae of SCNT cloned Korean native cattle that died immediately after birth and in normal placentae obtained by artificial insemination. Abnormal expression of these two genes reported to be partly responsible to for abnormal placental function and low survivability of cloned claves. Results from our study regarding these two gene showed that showed hypermethylation in gene region of *TIMP-2* gene and no methylation in gene regions of *SOD1* gene in cloned blastocysts relative to IVF counterparts. For *TIMP2* gene, our results are not in agreement with those of above study but our results for *SOD1* gene, are in agreement with above study as its unmethylated status can suggesting high level of gene expression.

Gene such as *AQP9* encode a water channels protein which is involved in coordinating urea transport. Gao et al.^58^ reported abnormally lower expression level of *AQP9* gene in placenta of SCNT cattle by both RNA-seq and q-PCR. Similarly, in present study, methylation level of *AQP9* gene was found to be very high which suggests lower expression level of this gene in cloned embryos relative to IVF counterparts. Sood et al.^56^ also reported down-regulated expression of *DKK3* gene in cloned embryos in comparison with IVF embryos. Likewise, our study showed high number of hypermethylated regions of *DKK3* gene in cloned blastocysts relative to IVF counterparts that might indicate towards low expression level of this gene. Together, this study suggests that cloned embryos and IVF embryos present a profound differences in global DNA methylation profile which is consistent throughout all developmental stages. These differences are related to distinct DMRs and CpGis distribution pattern, gene ontology terms and pathways in cloned embryos relative to IVF counterparts. A large number of pathways are found to be affected by hypermethylation in cloned relative to IVF embryos. Among these, the major pathways related to embryonic development are Integrin signalling pathway, Wnt signaling pathway, apoptosis signaling pathway, inflammation mediated by chemokine & cytokine signaling pathway, gonadotropin-releasing hormone receptor pathway, CCKR signaling map, angiogenesis. Further investigation is needed to understand the crosstalk among other epigenetic mechanisms that all together regulate these pathways.There is a need of further more targeted studies stimulated by this study to evaluate hypermethylation in exact genomic locations, promoters and gene body and role of regulatory proteins in establishing and eliminating methylation in the genome. It is essential to clarify whether this DNA hypermethylation alone is responsible for causing low efficiency of blastocyst rate and live birth rate of cloned offsprings and how approved epigenetic modifiers drugs can be used to prevent developmental failure of cloned embryos.

## Methods

### Ethics statement

All methods were carried out in accordance with guidelines of CPCSEA (Committee for the Purpose of Control and Supervision on Experiments on Animals) with due approval from the Institutional Animal Ethics Committee (IAEC) of ICAR-National Dairy Research Institute, Karnal, Haryana with approval number (F.No. 43-IAEC-18-36), dated 13.10.2018). The experimental animals used to obtain ovaries for this study were native Indian buffaloes breed ‘Murrah’. Efforts were made to minimize animals’ pain and suffering in abattoir at Delhi, India. Also, we tried to reduce the number of buffaloes required to complete this study.

### Sample collection

This study is based on buffalo embryos produced under in vitro conditions. No in vivo trials were carried out in any experiment. The present study involves two experimental groups’ i.e. cloned embryos and IVF embryos of buffalo. Cloned embryos were collected at different developmental stages (2-cell, 8-cell and blastocyst) at different interval post in vitro culture. Embryos produced by IVF (2-cell, 8-cell and blastocyst) were used as control in this study. Embryos used for global DNA methylation analysis were produced under same season to nullify effect of season variation.

The morphological and physiological evaluation was done before selecting oocytes. Compact cumulus-oocyte-complexes (COCs) with 2–8 mm diameter in size, unexpanded cumulus mass having ≥ 2 layers of cumulus cells, and with homogenous, evenly granular ooplasm were used for cloning and IVF purpose. Brilliant Cresyl Blue (BCB) stain was used to select oocytes with high developmental competence. After in vitro culture, cloned embryos at 2-cell, 8-cell and blastocyst stage were collected at 14–16 h_,_ 36–40 h and 8th day post in vitro culture (IVC), respectively as standardized in our lab. The embryos were washed thrice with DEPC-treated water and stored at − 196 °C in liquid nitrogen until further use. IVF embryos at 2-cell, 8-cell and blastocyst stage were collected at 24–26 h_,_ 68–72 h and 8th day post IVC, respectively^59^.

In this study, the total number of cell present in each pool of 2-cell, 8-cell and blastocysts stage produced by Hand-guided cloning and IVF was approximately 1000, 1000 and 6000 respectively for MeDIP-Seq over a particular season with 15 cloning trials and 10 IVF trials to avoid seasonal variations. For bisulfite sequencing PCR, blastocyst stage of cloned and IVF embryos were collected that contained approximately 6000 number of cells per pool. The donor fibroblasts cells used for HMC had been collected by biopsy of tail skin of bull No. Mu-4093, and were obtained from ICAR-Central Institute for Research on Buffaloes (CIRB), Hisar, in a cryopreserved form. Cryopreserved semen from the same bull (Mu-4093) was used for producing IVF embryos. Methods involved in vitro production of cloned and IVF embryos is described below.

### Chemicals

All the chemicals and media were purchased from Sigma- Aldrich Corp., (St. Louis, MO, USA), and all the plasticware was purchased from Becton, Dickinson and Co., (Lincoln Park, NJ, USA) or Nunc (Rosklide, Denmark) unless mentioned otherwise. Fetal bovine serum (FBS) was obtained from Gibco Life technology (Gaithersburg, MD, USA) whereas, Research Vitro Cleave medium (K-RVCL), was purchased from William A. Cook (Brisbane, Australia). In vitro maturation (IVM) and fertilization (IVF) of oocytes, in vitro culture (IVC) of presumptive zygotes produced by IVF and reconstructed embryos produced by HMC and culture of somatic cells were carried out in a CO2 incubator (5% CO2 in air, 90–95% relative humidity.

### In vitro embryo production by HMC and IVF

Buffalo ovaries from the slaughterhouse were aspirated for Cumulus-oocyte complexes (COCs). COCs were classified into useable and unusable categories based on their morphological appearance as ones with evenly granular homogenous ooplasm and unexpanded cumulus mass with ≥ 2 layers of cumulus cells are considered as useable. The oocytes which were wholly or partially denuded of the cumulus mass or which had expanded cumulus mass or irregular ooplasm were classified as non-useable. For selection of COCs of high developmental competence, those of useable category were stained with Brilliant Cresyl Blue (BCB), as described previously^45^. COCs with distinct blue colour (BCBþ) were washed several times with the IVM medium, which consisted of TCM-199 + 10% FBS þ 5 mg/mL porcine follicle stimulating hormone (pFSH) + l mg/mL estradiol-17β + 0.81 mM sodium pyruvate + 50 mg/mL gentamicin sulfate. For IVM, the COCs were placed in 100 mL droplets of the IVM medium (15–20 COCs per droplet), overlaid with sterile mineral oil in 35 mm Petri dishes and cultured in a CO^2^ incubator at 38.5 °C for 21 h as previously described^60,61^. Culture of donor fibroblast cells to full confluence to synchronize them in G1 stage of cell cycle and all other procedures of HMC were carried out as described previously^62^. For production of IVF embryos, oocytes were subjected to IVM for 24 h to achieve metaphase II stage with production of first polar body. IVF and IVC were carried out as described previously^63^. Semen of the same buffalo bull, the somatic cells of which had been used for HMC, was used for IVF for producing genetically half-identical IVF blastocysts.

### DNA extraction

Total DNA was isolated from respective pools of cloned and IVF embryos using Arcturus^®^ PicoPure^®^ DNA Extraction Kit (Arcturus, Mountain View, CA, USA, catalogue no.KIT0103) according to the manufacturer’s instructions. Briefly, each pool of embryos (2-cell, 8-cell and blastocysts) was transferred to 1.5 mL DEPC-treated tube and was pelleted by centrifugation. The supernatant was removed and the pellet was resuspended in 150 μL of Extraction Solution. The suspension was mixed well by vortexing for a few minutes. The samples were then incubated in a dry bath at 65  °C for 3 h. The sample tubes were then incubated in a dry bath at 95 °C for 10 min to inactivate Proteinase K. The purified DNA thus obtained is ready to be used for further processing or can be stored at − 20 °C. DNA quality control analysis was carried using Invitrogen™ Qubit™ 3 Fluorometer.

### Immunoprecipitation of genomic DNA

Methylated DNA Immunoprecipitation (MeDIP) Kit (Abcam-117133) was used according to manufacturer’s instructions to isolate methylated DNA from the embryo samples. Special low DNA LoBind tubes (Ep- pendorf #022431021) were used during the MeDIP procedure to prevent loss of DNA. DNA samples were sonicated with 3 pulses of 10–12 s each at level 2 using a Branson Microtip probe, followed by 30–40 s rest on ice between each pulse. Initially, sonicated DNA (25 ng from each pool) was diluted in 450 µL of TE buffer (10 mM Tris–HCl, 1 mM EDTA, pH 8.0) and then denatured at 95 °C for 10 min. A portion of the sonicated DNA was reserved as Input (control) and was not subjected to immunoprecipitation. The denatured DNA was placed on ice for 10 min and was then incubated with 5mC monoclonal antibody for 2 h at 4 °C on an orbital shaker (50–100 rpm). After immunoprecipitation, methylated DNA fragments were column purified and eluted in 20 µL of elution buffer and stored at − 20 °C till further used for library preparation.

### MeDIP-sequencing

#### Library preparation and quality control

Global DNA methylation analysis of Handmade cloned and IVF embryos at 2-cell, 8-cell and blastocyst stages was done using MeDIP-Seq by a commercial service provider, M/S DNA Xpert, New Delhi, India.

Illumina library preparation was performed by using the NEBNext Ultra DNA Library Prep Kit for Illumina (NEB, New England Biolabs Inc., USA #E7370S). Sonicated DNA (25 ng from each pool) was end repaired into dA-tailed fragments. Then the NEBNext-indexed adaptor was ligated into sonicated DNA fragments. Adaptor-ligated DNA was further cleaned up by AMPure XP beads (Beckman Coulter, Inc., Life Sciences, United States #A63881) and then subjected to the MeDIP procedure. The Input DNA was linked to different index primers. After immunoprecipitation, adaptor-ligated DNA was extracted by phenol/chloroform and precipitated by ethanol. The antibody-enriched DNA was subsequently amplified by using an index primer and universal PCR primer provided in NEB Multiplex Oligos for Illumina. PCR was performed with 20 cycles for each MeDIP sample. The PCR products were then purified by AMPure XP beads and eluted with TE buffer. The library concentration and size distribution was analyzed by Agilent 4200 Tapestation.The library was run on Agilent 4200 Tape Station and the resulting profile was evaluated. QC-passed libraries (peak size varies from 28 to 400 bp) were used to generate high-quality reads for further analysis.

#### Sequencing and quality control

The QC-passed were then set up on Illumina HiSeq 2500 platform for sequencing. Quality check of raw reads was carried out using FastQC (v0.11.5). Based upon Phred score value, all the reads were found to be of good quality, therefore, no filtering was required for any of the samples. The QC-passed high-quality reads (Phred Score Cut off of Q 20) were used for further analysis.

### MeDIP-Seq data analysis

R package MEDIPS. R (v3.6.3) and MEDIPS (v1.36.0) softwares was used for analysis of raw data. The MeDIP-Seq data analysis workflow employed in the present study consisted of following major steps viz., loading of raw data into MEDIPS software, quality check of data, identification of differentially methylated regions (DMRs) based upon fold change criteria (with length of DMR around 1999 bp in the program window) and classification/clustering of genes. The gene ontology (GO) enrichment analysis and pathway analysis was done by using R package MEDIPS and Panther Classification System, respectively. The reads generated were aligned using bowtie2 (v2.2.8) software to *Bos taurus* reference genome, UMD 3.1.1. CpG islands of genome were identified using cpgiscan (v1.0). CpGi annotation for each sample was done by intersecting bam file against the cpgiscan bed output using bedtools. Distribution of CpGs and feature annotation at different components of genome (promoter,exon, intron and intergenic region) was done by using R package genomation. genomation (v1.16.0). Distribution of CpG islands and their methylation status in different repetitive elements of genome (LINE, SINE, repetitive DNA, LTR, simple repeats, low complexity repeats) was done by using RepeatMasker.

### Bisulfite sequencing PCR analysis

Five gene regions were selected randomly to validate MeDIP-Seq data. For that, 25 blastocyst-stage for each cloned and IVF embryos were used for validation of MeDIP-Seq data by bisulfite sequencing PCR analysis. Genomic DNA was isolated from respective pools of cloned and IVF blastocyst-stage embryos using the DNeasy^®^ Blood & Tissue Kit (Qiagen, catalogue no. 69504) according to the manufacturer’s instructions. The purified DNA thus obtained is ready to be used for further processing or can be stored at − 20 °C. DNA quality control analysis was carried on NanoDrop™ 2000 (Thermo Scientific, USA). Bisulfite conversion of DNA samples isolated from cloned and IVF blastocyst-stage embryos was done using EZ DNA Methylation-Direct™ Kit (Cat. No. D-5020, Zymo Research, Orange, CA, USA) according to the instruction manual, with minor modifications as detailed; Genomic DNA isolated using above protocol was used for bisulfite conversion. DNA (20 μL) was added to 130 μL of CT Conversion Reagent solution and was centrifuged briefly to ensure that there were droplets in the cap or on sides of the tube. The mixture was then heated in a thermal cycler at 98 °C for 8 min and 64 °C for 3.5 h and was then stored at 4 °C for up to 20 h. Bisulfite converted DNA was desalted, purified and eluted with 40 μL of elution buffer. The O.D. of the bisulfite-converted DNA was recorded. Consequently, bisulfite sequencing PCR (BSP) was carried out with 1 μL of modified DNA per PCR reaction Meth-primers for selected five genes were designed from The Li Lab (MethPrimer) software at 2 kb upstream regions covering at least one CpG island. For designing bisulfite PCR primers, the sequences for each gene were taken from NCBI (http://www.ncbi.nlm.nih.gov). The sequences of the BSP primers used to amplify the targeted products are shown in (Supplementary Table 9). Hotstart PCR was carried out for bisulfite-converted DNA pairs using Hotstart DNA polymerase (Zymo Taq™ premix, Zymo Research). The amplified product was taken as template DNA (with 1:5 dilution) for second PCR amplification with the same primers. The PCR cycle included denaturation for 10 min at 95 °C followed by 35 repeated cycles of 95 °C for 30 s, annealing at variable temperatures for 30 s and extension at 72 °C for 30 s followed by a final extension at 72 °C for 10 min. Resulting PCR products were run on 2% agarose gel to confirm the amplicon size. Purification of the bisulfite-treated amplified product was done with GeneJET PCR Purification Kit (Thermo Fisher Scientific, Inc. USA #K0701). Purified PCR products were sent for custom sequencing to M/S DNA Xpert Pvt. Ltd., (New Delhi, India). The PCR products were sequenced in triplicates with both forward and reverse primers of selected region of interest. The data files generated with Sanger sequencing were processed using the Sequencing Analysis V5.3 software. Sequences with non-overlapping peaks are considered to be good for further analysis. Peaks with high background of < 50% of the co-efficient of variation (CV) are also considered to be good for further analysis. By using Seq Scanner, PDF and Fasta files were created from ABI files. Analysis of data generated for calculating methylation level (%) and creating lollipop diagrams for each sample was done by using BioEdit version 7.2.5 software.

## Supplementary Information

Supplementary Information.

## Data Availability

The high-throughput sequencing data (Raw data/ FastQ) have been submitted in NCBI’s Sequence Read Archive (SRA) under BioProject ID: PRJNA723972.
